# Safety and Tolerability of Tolvaptan in an Autosomal Dominant Polycystic Kidney Disease Spanish Cohort: A Real-World Experience

**DOI:** 10.7759/cureus.10207

**Published:** 2020-09-02

**Authors:** Xavier E Guerra-Torres, J Peña Esparragoza, M Perez Fernandez, M Fernandez Rodríguez, J Mancha Ramos, P Martinez Miguel, D Rodriguez Puyol, H Bouarich

**Affiliations:** 1 Nephrology, Hospital Universitario Príncipe de Asturias, Madrid, ESP

**Keywords:** autosomal dominant polycystic kidney disease, polycystic kidney disease, tolvaptan, v2-receptor antagonist

## Abstract

Introduction: Autosomal dominant polycystic kidney disease (ADPKD) is the commonest inherited disorder of the kidneys. A vasopressin V2-receptor antagonist (tolvaptan) was recently approved for the treatment of ADPKD. This study aims to analyze the safety and tolerability of tolvaptan for the management of ADPKD patients in a real-world setting.

Methods: We conducted a descriptive retrospective study in ADPKD patients in an outpatient clinic setting in Spain from 2018 to 2019. Descriptive statistical analysis of demographics and clinical data, at baseline and one year after tolvaptan initiation, was assessed. Data are presented as median and interquartile range, and as frequencies for categorical variables.

Results: Ten patients with ADPKD were identified. At baseline median age was 49.5 (38.5-63.5) years and 60% were males. During treatment with tolvaptan, no significant aquaresis-related symptoms or hepatotoxicity were described. No serious adverse events, discontinuation, or deaths were reported during the study.

Conclusion: Tolvaptan was well-tolerated without severe adverse events in patients with ADPKD who showed rapid disease progression criteria. Longer follow-up is required to learn about the long-term effects of this treatment.

## Introduction

Autosomal dominant polycystic kidney disease (ADPKD) is the most common inherited disorder of the kidneys. In Europe, ADPKD affects about one per 800-1000 people and accounts for 6-10% of end-stage renal disease (ESRD) patients [1-3]. ADPKD is characterized by mutations in polycystic kidney disease 1 (PKD1), polycystic kidney disease 2 (PKD2) and glucosidase II alpha subunit (GANAB) genes that increase the cyclic adenosine monophosphate (cAMP) intracellular levels and upregulate the mammalian target of rapamycin receptor (mTOR). These abnormalities result in the development and enlargement of kidney cysts and cell proliferation, leading to ESRD. Also, systemic clinical features like hypertension, cerebral aneurysm, liver cyst, and valvular heart disease are common [4-7].

Recently, tolvaptan, a vasopressin V2-receptor antagonist, was approved for the treatment of ADPKD [8,9]. Tolvaptan showed a reduction in kidney volume, decrease in the slope of serum creatinine and glomerular filtration rate worsening while reducing the frequency of ADPKD-related complications derived from large kidney volume. However, patients with ADPKD could have safety or tolerability concerns like increased thirst, concurrent comorbidities, major side effects leading to non-compliance with therapies, or taking therapies likely to interfere with tolvaptan.

This study aims to analyze the safety and tolerability in ADPKD patients attending our nephrology outpatient clinic who were treated with tolvaptan in the management of ADPKD.

## Materials and methods

A descriptive retrospective study was conducted in the Nephrology Section outpatient clinic of Principe de Asturias University Hospital (Alcalá de Henares, Spain) in 2018. All subjects with ADPKD that fulfilled the criteria for rapid disease progression, according to the Mayo Clinic Imaging classification, and were treated with tolvaptan were eligible [10]. Height-adjusted total kidney volume (hTKV) was assessed by abdominal magnetic resonance imaging (MRI) scan and measured by stereology. Imaging and clinical data were collected for each subject from the electronic medical record.

We performed a descriptive statistical analysis of demographic and clinical characteristics at baseline and one year after tolvaptan initiation. The analyzed variables were age, sex, comorbidities, estimated glomerular filtration rate (eGFR) calculated by Chronic Kidney Disease Epidemiology Collaboration (CKD-EPI) equation, chronic kidney disease staging, hTKV, Mayo Clinic Imaging classification for ADPKD, presence of complex or liver cyst, hematuria, kidney volume and drug-derived symptoms, history of nephrolithiasis, urinary tract infections (UTI) or intracerebral hemorrhage, tolvaptan vintage and dosage, hepatotoxicity, aquaresis-derived symptoms, and serum and urinary biochemistry. Statistical analysis was performed with the Statistical Product and Service Solutions (SPSS), version 22 (IBM Corp., Armonk, NY) software. Continuous variables are presented as median and interquartile range (IQR) and as frequencies for categorical variables.

## Results

In our study, 10 patients with ADPKD that fulfilled the criteria for rapid disease progression and were treated with tolvaptan were identified [10]. Using the Kolmogorov-Smirnov Z test, we found that continuous variables showed a non-normal distribution, whereby results are shown as medians and interquartile range (IQR). At baseline, median age was 49.5 (38.5-63.5) years and 60% were males. Demographic characteristics, clinical data, and main laboratory findings are summarized in Table 1.

**Table 1:** Clinical characteristics of ADPKD patients treated with tolvaptan at baseline and one year after treatment initiation. IQR: interquartile range; eGFR: estimated glomerular filtration rate; CKD-EPI: Chronic Kidney Disease Epidemiology Collaboration equation; SBP: systolic blood pressure; DBP diastolic blood pressure; ACEI: angiotensin-converting enzyme inhibitors; ARB: angiotensin II receptor blockers; CKD: chronic kidney disease; ADPKD: autosomal dominant polycystic kidney disease; hTKV: height adjusted total kidney volume; UTI: urinary tract infection; NC: not calculated; NA: not available in electronic medical record. ^†^ one-year after treatment initiation; ^§^ All patients started with tolvaptan 60 mg at baseline.

Patients Characteristics, n= 10	Baseline	Tolvaptan ^†^
Age (years), median (IQR)	49.5 (38.5-63.5)	NC
Male, n (%)	6 (60%)	6 (60%)
Height (m), median (IQR)	1.7 (1.6-1.8)	NC
eGFR (CKD-EPI) (mL/min/1.73 m^2^), median (IQR)	49.2 (38.7-57)	42.6 (28.6-63.8)
Hypertension, n (%)	8 (80%)	8 (80%)
SBP (mmHg)	127.5 (120-132)	125 (117.5-132.5)
DBP (mmHg)	80 (67.5-88.8)	75 (60-82.5)
ACEI	5 (62.5%)	5 (62.5%)
ARB	3 (37.5%)	3 (37.5%)
Diabetes mellitus, n (%)	1 (10%)	1 (10%)
CKD stage 2, n (%)	2 (20%)	2 (20%)
CKD stage 3, n (%)	7 (70%)	5 (50%)
CKD stage 4, n (%)	1 (10%)	3 (30%)
ADPKD vintage (years), median (IQR)	9 (2-21.5)	NC
hTKV (mL/m), median (IQR)	1361.1 (1165.5-1801)	NC
ADPKD Mayo Clinic Imaging classification 1C, n (%)	4 (40%)	NC
ADPKD Mayo Clinic Imaging classification 1D, n (%)	3 (30%)	NC
ADPKD Mayo Clinic Imaging classification 1E, n (%)	3 (30%)	NC
Complex cyst, n (%)	2 (20%)	2 (20%)
Hemorrhagic cyst, n (%)	2 (20%)	2 (20%)
Liver cyst, n (%)	8 (80%)	8 (80%)
Abdominal pain, n (%)	2 (20%)	2 (20%)
Hematuria, n (%)	1 (10%)	1 (10%)
UTI, n (%)	1 (10%)	1 (10%)
Tolvaptan dosage (mg), median (IQR)	60 ^§^	120 (60-120)
Down-titration of ACEI/ARB dose, n (%)	NC	2 (20%)
Serum Creatinine (mg/dL), median (IQR)	1.6 (1.3-2)	1.9 (1.2-2.3)
Albuminuria (mg/24h), median (IQR)	80 (29-408)	NA
Serum Sodium (mmol/L), median (IQR)	140.5 (139-143.3)	142 (139.8-146.3)
Plasma Osmolality (mOsm/Kg), median (IQR)	295.6 (292-300.2)	296.5 (288.9-312)
Urinary Osmolality (mOsm/Kg), median (IQR)	325 (311.8-353.3)	210.5 (126-282.3)
Serum Uric Acid (mg/dL), median (IQR)	6.5 (6.1-7.1)	7 (5.7-8.4)

Median of eGFR calculated by CKD-EPI equation was 49.2 (38.7-57) mL/min/1.73 m^2^ distributed in CKD stage 2, 3 and 4 (20%, 70% and 10% respectively). Hypertension was a common condition (80%), while diabetes was present in 10% of patients. The ADPKD diagnostic vintage was nine (2-21.5) years and hTKV, assessed by abdominal MRI scan, was 1361.1 (1165.5-1801) mL/m. According to hTKV, ADPKD Mayo Clinic Imaging classification was 1C (40%), 1D (30%) and 1E (30%), showing criteria for rapid disease progression. ADPKD patients showed complex cysts in 20% of cases, hemorrhagic cysts in 20%, and 10% of patients had macroscopic hematuria. Kidney volume-derived symptoms as abdominal pain were found in 20% of patients. History of nephrolithiasis or intracerebral hemorrhage were negative; UTI was present in one patient. The median treatment vintage was five (1-12) months and 60% of patients reached a maximum dose of tolvaptan (120 mg/day). Angiotensin-converting enzyme inhibitors (ACEI) and angiotensin II receptor blockers (ARB) were prescribed in 62.5% and 37.5% of hypertensive patients, respectively. Blood pressure marginally decreased after one year of treatment and a down-titration of antihypertensive medication was necessary in two patients. During treatment with tolvaptan, no relevant aquaresis-related symptoms (polyuria/polydipsia), hepatotoxicity or hyperuricemia (or gout events) were reported. Serum liver enzymes remained close to normal range during follow-up. Only mild hypernatremia was found in one case. No serious adverse events, discontinuations, or deaths were reported during the study.

## Discussion

In our descriptive analysis, patients with ADPKD treated with tolvaptan had good tolerability with no clinically relevant side effects after one year of treatment. Two main clinical trials have been performed to test tolvaptan efficacy and safety in the management of ADPKD, the Tolvaptan Efficacy and Safety in Management of Autosomal Dominant Polycystic Kidney Disease and Its Outcomes (TEMPO) 3:4, and the Replicating Evidence of Preserved Renal Function: An Investigation of Tolvaptan Safety and Efficacy (REPRISE) studies [8,9]. In brief, TEMPO 3:4 was a phase 3, multicenter, double-blind, placebo-controlled trial, that randomly assigned 1445 ADPKD patients, 18 to 50 years of age with a total kidney volume ≥750 mL, eGFR (CKD-EPI) ≥60 mL/min/1.73 m^2 ^in a 2:1 ratio to receive tolvaptan or placebo for 36 months. The REPRISE study involved 1370 ADPKD patients in a phase 3, multicenter, placebo-controlled, double-blind trial with an eGFR (CKD-EPI) between 25 to 65 mL/min/1.73 m^2^ (for subjects aged in 18 to 55 years) or 25 to 44 mL/min/1.73 m^2^ (for subjects aged in 56 to 65 years) in a 1:1 ratio to receive tolvaptan or placebo for 12 months.

In TEMPO 3:4, adverse events were similar in the tolvaptan group (98%) and placebo (97%). The tolvaptan group had a higher frequency of aquaresis-derived adverse events, whereas the placebo group had higher frequencies of volume or cyst-derived adverse events (abdominal pain, hematuria, and UTI). Patients receiving tolvaptan showed a higher rate of hepatotoxicity and hypernatremia than patients in the placebo group, with a discontinuation rate of 8% and 1%, respectively. Frequency of gout was around 3% in the tolvaptan group compared to 1% in the placebo group. No mortality associated with tolvaptan use was reported during the trial [8].

In the REPRISE study, rates of adverse events did not differ between tolvaptan (85%) and placebo (82%) groups during the double-blind period. Similar to TEMPO 3:4 trial, in the REPRISE study the tolvaptan group had a higher frequency of aquaresis-derived symptoms, whereas the placebo group had higher frequencies of abdominal pain and UTI. Discontinuation due to adverse events was 10% in patients receiving tolvaptan, compared to 2% in the placebo group. Finally, abnormalities in liver enzymes had a frequency of 11% in the tolvaptan group compared to 5% in patients receiving placebo. No death associated with tolvaptan use was reported during the trial [9].

Major strengths of our analysis are the fact that is a real-world experience in patients with ADPKD with the comorbidities and peculiarities of our population, including subjects with an eGFR below 30 mL/min/1.73 m^2^. Besides these strengths, key limitations of our study are a small sample size in a retrospective analysis with a limited follow-up period.

## Conclusions

In conclusion, our study shows that tolvaptan was well-tolerated without clinically relevant aquaresis-related symptoms, hepatotoxicity, hyperuricemia, or gout events after one year of treatment in patients with ADPKD who showed rapid disease progression criteria. Also, no severe adverse events or death during the study were reported. Longer follow-up is required to learn about long-term effects of this treatment.
